# Supplementary material for: Multi-objective QSAR prediction of ERα antagonists via SHAP-based interpretation
Source: PLoS One. 2026 Jan 8;21(1):e0338080. doi: 10.1371/journal.pone.0338080 (PMC12782375; doi:10.1371/journal.pone.0338080)
Supplement: Attachment — Submitted filename: PONE-D-25-46112.docx [file pone.0338080.s001.docx]

The manuscript titled **"*Multi-objective QSAR Prediction of ERα Antagonists via SHAP-based Interpretation*"** proposes a two-stage predictive framework combining QSAR and machine learning to evaluate both the biological activity and ADMET properties of drug candidates. While the study provides interesting results, several aspects require improvement, including:

- The article states that the dataset was obtained from the **ChEMBL** database, containing 1 974 compounds targeting ERα. However, it would be useful for the authors to **justify the choice of this specific database**, as such justification would strengthen the relevance and credibility of the study.
- A comparison with other **feature selection methods** would further validate the proposed DFFS approach.
- The discussion could benefit from being more closely linked to **previous studies**, to better contextualize the results.
- It is recommended to **improve the readability and quality of figures**.
- It would be useful for the authors to **include a dedicated section on the study’s limitations**, discussing methodological constraints.
- Although the metrics are defined, it would be helpful to **explain what typical values represent** for these metrics (e.g., what values indicate a good model in this context).
- The abstract mentions that high **pIC_50_** values indicate good **binding affinity** to the ERα receptor. However, it would be valuable to **directly evaluate the binding affinity** of the high-activity candidate compounds, for example through **ΔG binding calculations or molecular docking studies**. This would help validate and complement the bioactivity predictions by providing a quantitative measure of the ligand-receptor interaction.
- Information included on the different modeling techniques, QSAR and ADMET, is quite trivial and should be more detailed. To enhance the introduction of the computational section, it is recommended to provide a detailed discussion as reported in the literature. In order to facilitate a better understanding for readers, the following previous studies employing docking and molecular dynamic in this research area can be cited: [10.1002/jccs.202200195](https://doi.org/10.1002/jccs.202200195); [10.1016/j.molstruc.2022.133964](https://doi.org/10.1016/j.molstruc.2022.133964);

[10.1016/j.jtusci.2015.04.007](https://doi.org/10.1016/j.jtusci.2015.04.007); [10.1021/acsomega.2c07585](https://doi.org/10.1021/acsomega.2c07585); [10.1002/slct.202301092](https://doi.org/10.1002/slct.202301092); [10.1016/j.chphi.2023.100361](https://doi.org/10.1016/j.chphi.2023.100361); [10.1016/j.chphi.2023.100361](https://doi.org/10.1016/j.chphi.2023.100361); [10.1155/2016/5137289](https://doi.org/10.1155/2016/5137289)
