# Supplementary material for: Multi-objective QSAR prediction of ERα antagonists via SHAP-based interpretation
Source: PLoS One. 2026 Jan 8;21(1):e0338080. doi: 10.1371/journal.pone.0338080 (PMC12782375; doi:10.1371/journal.pone.0338080)
Supplement: Attachment — Submitted filename: Response to Reviewers.docx [file pone.0338080.s003.docx]

Reviewer #1
Comment 1. The manuscript mentions 1,974 compounds retrieved from ChEMBL, but it is unclear whether activity values were standardized across assay types (e.g., binding affinity vs. functional assays). Please clarify the inclusion/exclusion criteria for compounds and how assay heterogeneity was handled, as inconsistent experimental data can bias QSAR models.

Re: We sincerely appreciate the reviewer’s constructive comments. Regarding the concern about potential experimental heterogeneity in the activity data, we would like to clarify that all data used in this study were obtained from the publicly available standard dataset released by the 18th China Postgraduate Mathematical Contest in Modeling ([www.shumo.com/wiki/doku.php](http://www.shumo.com/wiki/doku.php)). Before being made public, this dataset underwent rigorous preprocessing by the organizers, including experimental data cleaning, unit harmonization, and standardization across different experimental types. These steps effectively minimized potential biases arising from variations in experimental conditions, ensuring the consistency and comparability of the data. Given the academic authority and data credibility of the contest platform, the activity values and ADMET property data provided in this dataset are highly reliable and fully meet the scientific requirements for subsequent QSAR modeling. Furthermore, we have verified that the same dataset was also adopted in a study published in *Expert Systems with Applications*, titled “Screening model of candidate drugs for breast cancer based on ensemble learning algorithm and molecular descriptor” [DOI: 10.1016/j.eswa.2022.119185]. This publication explicitly stated that the data were derived from the ChEMBL database, further confirming the reliability of the dataset used in our research.

Comment 2. High AUC scores are reported, but no independent external validation set is used. This raises concerns about model generalizability beyond the training distribution.

Re: We sincerely appreciate the reviewer’s valuable comments on the generalization performance of our model. In response to the concern regarding the setup of the external validation set, we provide the following clarification. In the ADMET property modeling section, a stratified random sampling strategy was employed. Specifically, the entire dataset was first divided into a training set and an external test set at a ratio of 4:1. The test set was not involved in any feature selection, model training, or hyperparameter tunin and used solely at the final stage to evaluate the model’s generalization ability During model training, the training set was further split into two subsets at a ratio of 4:1, followed by five-fold cross-validation. In each fold, the model was independently optimized on different training subsets and evaluated on the corresponding validation subsets. This procedure effectively reduced random bias introduced by specific data partitions. The final performance metrics were obtained by averaging the results across the five folds, yielding a more generalized and reliable evaluation of model performance. In the revised manuscript, we have added a detailed description of the data partitioning and validation procedure in the methodological section corresponding to *Fig. 1 “Two-Stage Target Prediction Flowchart”*.

Comment 3. The Phi coefficient correlation analysis among ADMET properties is interesting, but the discussion risks over-interpretation without biological literature support.

Re: We sincerely appreciate the reviewer’s thoughtful concern regarding the potential risk of overinterpretation in the Phi coefficient correlation analysis. We have strived to interpret all correlation patterns objectively from the perspective of empirical data, avoiding speculative or overstated inferences. We have reviewed and integrated existing literature in the fields of drug discovery and ADMET optimization to provide biologically plausible explanations and supporting evidence for the observed statistical relationships. The detailed clarifications are as follows:

1. Negative correlation between Caco-2 permeability and hERG inhibition

We observed a significant negative correlation between intestinal permeability (Caco-2) and hERG channel blockade risk. This finding is supported by multiple lines of evidence. hERG channel blockade is recognized as a major cause of drug-induced cardiotoxicity during post-market surveillance, leading to QT interval prolongation—a severe and potentially fatal cardiac side effect [DOI: 10.1021/acs.jcim.1c00744; DOI: 10.1186/s12859-019-2814-5]. ADMET properties are not sequential processes but represent an integrated framework that considers interactions among absorption, distribution, metabolism, excretion, and toxicity [DOI: 10.1039/C8MD00472B; DOI: 10.1017/S0033583515000190]. The observed negative correlation suggests that compounds with favorable intestinal absorption may possess structural features that reduce their propensity to block the hERG channel—an important consideration in early-stage drug discovery.

2. Negative correlation between Caco-2 permeability and CYP3A4 metabolism, and positive correlation between Caco-2 and HOB

The negative correlation between Caco-2 permeability and CYP3A4 metabolism aligns with the well-established role of intestinal CYP3A4 in first-pass metabolism. The presence of CYP3A in the small intestine reduces the bioavailability of many orally administered drugs [DOI: 10.1124/dmd.105.008672]. CYP3A4 and CYP3A5 metabolize a large proportion of therapeutic agents and play a particularly important role in drug metabolism [DOI: 10.1016/j.pharmthera.2012.12.007; DOI: 10.7717/peerj.18636]. This relationship reflects an inherent trade-off in drug design: compounds engineered for high permeability may be more susceptible to extensive first-pass metabolism, ultimately reducing their oral bioavailability.

3. Positive correlation between hERG inhibition and CYP3A4 substrate liability

The observed positive correlation between hERG blockade risk and CYP3A4 substrate risk is consistent with the overlapping chemical space of these two properties. CYP3A4 is one of the most important enzymes in the cytochrome P450 family, responsible for metabolizing approximately 90% of marketed drugs [DOI: 10.1016/j.pharmthera.2012.12.007]. Both hERG and CYP3A4 tend to interact with lipophilic, aromatic, and basic compounds [DOI: 10.1021/acs.jcim.1c00744; DOI: 10.1016/j.ejmech.2020.112290]. This correlation underscores the importance of integrated ADMET optimization strategies in drug development, as mitigating one risk may inadvertently influence another.

4. Independence of micronucleus (MN) genotoxicity

We found no significant correlation between MN genotoxicity and other ADMET properties (CYP3A4, hERG, HOB), which is biologically meaningful. Inappropriate ADME characteristics and drug-induced toxicities are major factors preventing many early candidates from reaching the market [DOI: 10.1017/S0033583515000190; DOI: 10.2174/092986706778201558]. Genotoxicity mechanisms differ fundamentally from pharmacokinetic and metabolic properties, involving DNA damage pathways rather than enzymatic or receptor-mediated interactions. This independence highlights the necessity of evaluating genotoxicity as a distinct endpoint in drug safety assessment.

Comment 4. The threshold of pIC50 > 6 for active compounds should be pharmacologically justified, as the choice may influence screening outcomes.

Re: We have carefully re-examined the pharmacological rationale, literature evidence of this threshold, and we provide a detailed clarification below.

1. Flexibility and applicability of the threshold selection

pIC50 is defined as pIC50 = –log(IC50). Therefore, a pIC50 value of 6 corresponds to an IC50 of 10⁻⁶ M = 1 μM = 10³ nM. This logarithmic transformation is a common approach for assessing potency across orders of magnitude and enables consistent comparison among compounds. We acknowledge that different research stages and objectives may require different potency cutoffs. For instance, during hit identification, thresholds within the range of 1–10 μM are generally acceptable, whereas lead optimization often targets stricter standards such as 100 nM (pIC50 = 7) or lower [DOI: 10.1038/nrd3367]. Since the present study focuses on early screening and hit identification, adopting pIC50 ≥ 6 (IC50 ≤ 1 μM) represents a balanced and rational compromise—consistent with pharmacological meaning, literature precedent, and the goals of this study—while also ensuring appropriate sample distribution and modeling stability.

2. Pharmacological rationale and literature support

Using IC50 = 1 μM as the cutoff for identifying active or hit compounds is widely accepted in drug discovery. In early-stage screening, compounds with IC50 ≤ 1 μM are typically regarded as promising starting points for further optimization, whereas weaker compounds often lack sufficient potency or selectivity to advance into the lead optimization stage [DOI: 10.1111/j.1476-5381.2010.01127.x; DOI: 10.1038/nrd3368; DOI: 10.1038/nrd1086]. Numerous QSAR and virtual screening studies have adopted IC50 ≤ 1 μM as the activity threshold to construct binary classification models or primary filters—for example, *MATH: A Deep Learning Approach in QSAR for ERα Inhibitors* [DOI: 10.3390/molecules28155843] and *Exploring the Chemical Space of CYP17A1 Inhibitors Using Cheminformatics and Machine Learning* [DOI: 10.3390/molecules28041679]. These precedents provide empirical justification for using this biologically meaningful and operationally practical threshold in early-stage screening.

3. Methodological justification

To balance screening sensitivity, downstream validation cost, and model training quality, our study employed a two-stage sequential strategy. In the first stage (activity screening), a QSAR model was used to classify compounds as *Active* (pIC50 ≥ 6) or *Inactive* (pIC50 < 6), which aligns with established practices in high-throughput screening (HTS) and hit identification [DOI: 10.1038/nrd3367]. In the second stage (ADMET evaluation), only compounds classified as Active were subjected to further ADMET property prediction (Caco-2, CYP3A4, hERG, HOB, MN). This approach ensures that computational and experimental resources are focused on the most promising candidates, improving discovery efficiency and reducing redundant computation [DOI: 10.1016/j.drudis.2017.08.010; DOI: 10.1038/s41573-019-0024-5]. From a methodological standpoint, this cascaded strategy reduces resource waste and enhances data balance during model training. In our dataset, using 1 μM as the threshold yielded an *Active* vs. *Inactive* distribution of 1,231 vs. 743 samples, which helps prevent severe class imbalance and supports more robust model learning.

Comment 5. The discussion of limitations is rather brief. Points such as imbalanced ADMET datasets, exclusion of 3D descriptors, and the absence of docking/molecular dynamics validation should be more explicitly acknowledged to provide transparency for future improvements.

Re: We sincerely appreciate the reviewer’s valuable comments regarding the discussion of our study’s limitations. In the revised manuscript, we have expanded this section to provide a more comprehensive analysis and to outline clear directions for future improvement.

First, regarding the issue of class imbalance in the ADMET binary classification datasets, we fully recognize its potential impact on model training and evaluation. Although the sample distribution exhibited a certain degree of imbalance, the disparity was relatively moderate. Considering that the present study emphasizes data authenticity and representativeness, no data balancing techniques were applied during model training. We also attempted to employ the Synthetic Minority Oversampling Technique (SMOTE) to mitigate potential bias caused by class imbalance; however, the improvement in model performance was marginal, suggesting that SMOTE may have limited applicability under the current data scale and distribution characteristics. To address this issue, future studies will explore more advanced data augmentation strategies and cost-sensitive learning approaches to enhance the model’s adaptability to imbalanced data from an algorithmic perspective. In parallel, we plan to further expand the experimental dataset to improve the model’s representativeness and generalization across a broader chemical space.

Second, this study primarily utilized two-dimensional (2D) molecular descriptors for feature extraction and did not include three-dimensional (3D) descriptors. We recognize that 3D descriptors capture spatial conformations, electronic distributions, and stereochemical interactions between ligands and receptors with greater biological relevance—information that is often simplified or omitted in 2D representations. Therefore, future studies will integrate 3D molecular features with deep learning–based representations to enhance the model’s structural recognition capability, predictive accuracy, and interpretability.

Furthermore, in response to the reviewer’s suggestion regarding molecular docking and molecular dynamics validation, we have supplemented our revised study with molecular docking analyses. Specifically, we performed activity predictions on an additional independent dataset of 50 compounds and selected the top five candidates with the highest predicted activity for docking against the ERα target protein. The results showed that all selected compounds exhibited binding free energies (ΔG) below −7.0 kcal/mol, consistent with the model’s predictions, thereby providing static-level validation of the model’s rationality and reliability.

Finally, we acknowledge that molecular dynamics (MD) simulation has not yet been conducted, which remains an important limitation of the present study. Unlike docking, which provides a static evaluation of ligand–receptor binding, MD simulation can capture the dynamic behavior of complexes over time, yielding insights into the binding mechanisms under more physiologically relevant conditions. In future work, we plan to perform MD simulations on key compound–ERα complexes to analyze the time-dependent energy evolution and conformational stability of the binding systems. This will allow us to elucidate the dynamic characteristics of the ERα binding pocket and the conformational basis of bioactivity, thereby deepening the mechanistic interpretation and biological credibility of our predictive models.

Comment 6. SHAP analysis identifies key descriptors (LipoaffinityIndex, MDEC-23), but the mechanistic links to ERα antagonism need stronger justification with case-based examples.

Re: We sincerely appreciate the reviewer’s valuable comments regarding the mechanistic interpretation of the two key molecular descriptors, LipoaffinityIndex and MDEC-23, in relation to the antagonistic mechanism of ERα. In the revised manuscript, we have expanded our discussion to clarify the structural basis and logical rationale underlying their biological relevance.

Our analysis indicates that LipoaffinityIndex is one of the most influential descriptors for predicting pIC50. When LipoaffinityIndex exceeds 8, compounds generally exhibit higher biological activity. This descriptor quantifies a compound’s affinity for lipid environments, closely associated with membrane permeability and cellular uptake efficiency. Lipophilicity plays a critical role in the mechanism of ERα antagonists, which must traverse the cellular membrane to reach the intracellular receptor target [DOI: 10.1038/39645]. Higher lipophilicity facilitates passive diffusion across the lipid bilayer, allowing effective intracellular exposure even at lower extracellular concentrations, thereby improving apparent potency (pIC50) [DOI: 10.1016/s0169-409x(00)00129-0]. Recent studies on selective estrogen receptor modulators (SERMs) further confirm this trend, suggesting that optimal lipophilicity represents a balance between membrane permeability and receptor-binding affinity [DOI: 10.1016/j.pharmthera.2017.12.012; DOI: 10.1158/1078-0432.CCR-15-0360]. Computational chemistry studies have also demonstrated that moderate increases in lipophilicity significantly enhance cellular uptake and pharmacological activity of ERα antagonists, with LogP values in the range of 4–7 typically representing the optimal balance [DOI: 10.1021/acs.jmedchem.6b01355; DOI: 10.2133/dmpk.19.327].

Meanwhile, our results show that MDEC-23 exhibits a distinct threshold-dependent relationship with compound inhibitory activity: when MDEC-23 < 20, pIC50 is negatively correlated with MDEC-23, whereas this relationship becomes positive when MDEC-23 > 20. MDEC-23 characterizes the topological distance between secondary and tertiary carbon atoms, reflecting the spatial extension and flexibility of the molecular skeleton. Lower MDEC-23 values correspond to compact, rigid frameworks that may enhance intramolecular stability but limit conformational adaptability and induced-fit interactions with the receptor [DOI: 10.2174/1574884711308020006]. Conversely, higher MDEC-23 values indicate greater structural flexibility and spatial reach, enabling the molecule to adopt multiple conformations during binding and better accommodate the receptor’s dynamic cavity. Structural biology studies have shown that the ERα ligand-binding pocket has a volume of approximately 450 Å³ and accommodates ligands through an induced-fit mechanism that allows conformational adaptation [DOI: 10.1021/bi901149t; DOI: 10.1016/j.molcel.2015.01.025; DOI: 10.7554/eLife.12792]. Molecular dynamics simulations further reveal that molecules with higher MDEC-23 values possess extended backbones capable of interacting simultaneously with residues in both agonist and antagonist regions, thereby stabilizing the receptor’s antagonistic conformation by preventing the repositioning of helix H12 [DOI: 10.1016/j.drudis.2016.11.001; DOI: 10.3389/fphar.2018.00492]. Thus, an MDEC-23 threshold around 20 may represent the minimal structural extension required for effective cross-domain interactions. This finding aligns with recent structure–activity relationship (SAR) studies, which suggest that molecules with moderate flexibility and spatial extension are more likely to establish stable ERα antagonist binding modes and effectively block coactivator recruitment [DOI: 10.1158/2159-8290.CD-17-1229; DOI: 10.1158/0008-5472.CAN-15-2357].

In summary, we have elucidated the biophysical and structural mechanisms underlying the roles of LipoaffinityIndex and MDEC-23, reinforcing the structural rationality and interpretability of our SHAP-based findings. These explanations have been incorporated and refined in the revised manuscript, specifically in the discussion of *Fig. 5 “SHAP Scatter Plot for pIC50.”*

Comment 7. The study lacks benchmarking against recent state-of-the-art QSAR/ML or ChemBERTa-based models for ERα prediction, limiting claims of novelty.

Re: We sincerely appreciate the reviewer’s constructive comments regarding the model’s innovativeness and the need for comparisons with advanced deep learning approaches. In the revised manuscript, we have incorporated new experiments based on the ChemBERTa molecular language model and compared their results with those of our original LightGBM model. The detailed explanation is as follows.

1. Two-stage workflow of ChemBERTa modeling

Stage I – Feature extraction: We employed the pre-trained model ChemBERTa-100M-MLM from the HuggingFace platform to encode the SMILES sequences of all compounds. Each SMILES string was normalized into a token sequence of length 150, producing embedding tensors of shape [1974, 150, 768]. Through molecular language modeling, ChemBERTa learns high-dimensional semantic representations that effectively capture structural patterns, functional group combinations, and electronic effects within molecules. These embeddings can be directly used for downstream regression or processed via the [CLS] representation or mean pooling to support nonlinear learning of the structure–activity relationship [DOI: 10.1186/s13321-024-00929-7]. Among the ChemBERTa variants (77M, 10M, and 5M), the 100M parameter model demonstrates superior semantic richness and expressive capacity, providing a more comprehensive molecular representation for QSAR modeling.

Stage II – Downstream regression task: The dataset of 1,974 molecular samples was split into a training set and test set at a 4:1 ratio. Two representative models were then constructed: (1) the LightGBM model based on two-dimensional molecular descriptors (as in the submitted manuscript), and (2) a multilayer perceptron (MLP) model using ChemBERTa embeddings. For LightGBM regression, all features were normalized twice via MinMaxScaler, followed by PCA dimensionality reduction to 97 principal components (cumulative variance explained: 95%), preserving key structural information while reducing noise and redundancy. Hyperparameters were optimized using Hyperopt with five-fold cross-validation, minimizing RMSE as the objective function. The optimal parameters were: boosting_type = 'gbdt', num_leaves = 50, learning_rate = 0.06, max_depth = 8, bagging_fraction = 0.8, feature_fraction = 0.6, lambda_l1 = 0.4, lambda_l2 = 0.4, and num_boost_round = 750. For the MLP regression task, an end-to-end deep learning framework was established. The ChemBERTa embeddings were first flattened and then passed through three fully connected layers (512 → 256 → 128 units). Each layer was followed by Batch Normalization, ReLU activation, and Dropout (rate = 0.3) to improve training stability and generalization performance.

2. Comparative performance results

Table 1. Performance comparison of ChemBERTa-based models

| Dataset | MRE | MSE | MAE | R² |
| --- | --- | --- | --- | --- |
| Training set-LightGBM | 0.0106 | 0.0099 | 0.0671 | 0.9951 |
| Test set-LightGBM | 0.1032 | 0.7045 | 0.6507 | 0.6338 |
| Training set-MLP | 0.0456 | 0.1542 | 0.2954 | 0.9238 |
| Test set-MLP | 0.1011 | 0.7703 | 0.6365 | 0.6198 |

The predictive performance of ChemBERTa-based models is summarized in Table 1. Both LightGBM and MLP models achieved high training R² values (>0.9), but test-set R² values were only slightly above 0.6, suggesting mild overfitting. According to prior studies [DOI: 10.1002/minf.201000061; DOI: 10.1039/C7RA10979B], a test-set R² ≥ 0.6 generally indicates acceptable predictive performance for ERα inhibitory QSAR models, suitable for drug activity prediction and virtual screening tasks.

To further assess comparative performance, we contrasted ChemBERTa-based models with our original DFFS + LightGBM approach built on two-dimensional molecular descriptors. As shown in Table 2, the DFFS-LightGBM model achieved an R² of 0.7471, which is significantly higher than that of ChemBERTa-LightGBM (0.6338) and ChemBERTa-MLP (0.6198). This indicates that under a limited sample size, traditional descriptor-based models still provide superior physicochemical interpretability and structure–activity mapping capability.

Table 2. Comparative performance across different modeling strategies

| Method | MRE | MSE | MAE | R² |
| --- | --- | --- | --- | --- |
| DFFS-LightGBM | **0.0775** | **0.4865** | **0.4959** | **0.7471** |
| ChemBERTa-LightGBM | 0.1032 | 0.7045 | 0.6507 | 0.6338 |
| ChemBERTa-MLP | 0.1011 | 0.7703 | 0.6365 | 0.6198 |

In summary, the ChemBERTa model effectively captures high-dimensional semantic representations of molecular structures, confirming the potential of deep representation learning for modeling ERα inhibitory activity. However, given the current dataset size, the traditional LightGBM model based on molecular descriptors exhibits superior predictive stability and interpretability. Accordingly, we have systematically integrated the complete workflow and comparative results into the revised manuscript under the section *“Screening Model Based on Activity Prediction”*, to substantiate our analytical conclusions and highlight the scientific rationale behind the modeling strategy.

Comment 8. The Dual-Filter Feature Selection (DFFS) method is novel, but its stability should be validated across cross-validation folds or bootstrap sampling to ensure reproducibility of selected descriptors.

Re: We sincerely appreciate the reviewer’s attention to the stability and reproducibility of the DFFS method. To ensure that the features selected by DFFS possess both statistical robustness and methodological reproducibility, we implemented a multi-level control and validation framework throughout the design and experimental evaluation process.

First, during the feature importance assessment stage, DFFS integrates and quantifies descriptor importance through a fusion of MI, RF, and XGBoost algorithms. In the training phase, RF employs bootstrap sampling by default to enhance sample diversity, while XGBoost introduces controllable stochasticity at both the feature and sample levels through the parameters colsample_bytree (tree-level feature sampling), colsample_bynode (node-level feature sampling), and subsample (sample-level sampling). This multi-algorithm randomization strategy effectively enhances the robustness of feature selection. In addition, the entire modeling workflow adopts a five-fold cross-validation scheme, ensuring that consistent sets of key features can be obtained across different data splits.

Table 3. Comparative performance of different feature selection methods

| Feature Selection Method | MRE | MSE | MAE | R² |
| --- | --- | --- | --- | --- |
| DFFS-LightGBM | **0.0775** | **0.4865** | **0.4959** | **0.7471** |
| MI-LightGBM | 0.0788 | 0.5026 | 0.5028 | 0.7388 |
| XGBoost-LightGBM | 0.0818 | 0.5254 | 0.5226 | 0.7269 |
| SHAP-LightGBM | 0.0811 | 0.4908 | 0.5183 | 0.7449 |

Second, to further verify the effectiveness and stability of the DFFS method, we compared the DFFS-selected features with those obtained using individual feature selection methods—namely, MI, XGBoost, and XGBoost-SHAP. All experiments used LightGBM as the downstream predictive model to maintain comparability. As shown in Table 3, DFFS-LightGBM achieved superior performance across all evaluation metrics (R², MRE, MSE, and MAE) compared with single-method approaches. These results demonstrate that by integrating multiple feature selection strategies, DFFS can more robustly capture the complex associations between molecular structures and biological activity.

Under various cross-validation folds and random sampling conditions, DFFS consistently exhibited strong stability and reproducibility, demonstrating enhanced robustness of the feature selection outcomes. We have added the corresponding experimental procedures and result descriptions in the revised manuscript under the section *“Screening Model Based on Activity Prediction”* to provide a clearer explanation of these findings.

Reviewer #2

Comment 1. The article states that the dataset was obtained from the ChEMBL database, containing 1,974 compounds targeting ERα. However, it would be useful for the authors to justify the choice of this specific database, as such justification would strengthen the relevance and credibility of the study.

Re: We sincerely thank the reviewer for the insightful comments regarding the rationale behind our selection of the ChEMBL database. A detailed explanation is provided below.

1. Academic Authority and Open-Access Nature of the ChEMBL Database

The ChEMBL database, maintained by the European Molecular Biology Laboratory–European Bioinformatics Institute (EMBL–EBI), is one of the world’s most influential open-access repositories of bioactivity data. Since its establishment in 2009, ChEMBL has become a core infrastructure in the fields of drug discovery and computational toxicology. In 2022, it was officially recognized as part of the first cohort of Global Core Biodata Resources (GCBRs), signifying that its data curation standards and quality-control systems have undergone rigorous evaluation by the international life science community [DOI: 10.1093/nar/gkad1004]. ChEMBL is distributed under the Creative Commons Attribution–ShareAlike 3.0 license, ensuring full data accessibility, verifiability, and reproducibility, and thereby mitigating the reproducibility issues often associated with proprietary datasets.

Over the past decade, ChEMBL has become the de facto standard data source for academic and industrial research, supporting a wide range of applications from QSAR modeling to generative molecular design. Numerous high-impact studies have demonstrated its versatility—for example, in drug repurposing and molecular generation tasks [DOI: 10.1016/j.apsb.2022.11.010; DOI: 10.1016/j.mtbio.2022.100332; DOI: 10.1021/acscentsci.4c01991]. In parallel, frontier studies have integrated ChEMBL with other databases to expand chemical space and validate model generalization. The Macformer model reported in *Nature Communications* achieved outstanding performance across both internal ChEMBL and external ZINC datasets via reinforcement learning [DOI: 10.1038/s41467-023-40219-8]. Similarly, a *JACS* study demonstrated that multi-database pretraining on ChEMBL, ZINC, and COCONUT significantly enhances molecular diversity and chemical validity in generative models [DOI: 10.1021/jacs.4c08866]. Furthermore, a *Genome Medicine* publication showed how integrating ChEMBL’s chemical features with disease–gene networks (Enrichr, CTD, MSigDB) can improve drug–disease association mapping [DOI: 10.1186/s13073-025-01431-x].

2. Data Quality Control and Alignment with Research Objectives

The core strength of ChEMBL lies in its multi-level manual curation, structured data management, and standardized processing pipelines, which collectively ensure the accuracy, consistency, and reliability of molecular and bioactivity data [DOI: 10.1093/nar/gkw1074].
All experimental entries are manually extracted from peer-reviewed journals and patent literature (e.g., *J. Med. Chem.*, *Bioorg. Med. Chem. Lett.*), followed by multi-step expert verification to correct structural misannotations, unit discrepancies, and duplicate records [DOI: 10.1093/nar/gky1075]. Target annotations (e.g., UniProt accession) are precisely matched to ensure the accuracy of compound–receptor relationships. In terms of data standardization, ChEMBL systematically records experimental parameters such as cell lines, assay types, pH, and temperature, and harmonizes activity metrics (e.g., IC50, EC50) through unit normalization and value standardization. Molecular structures are preprocessed through salt stripping, stereochemical correction, and Standard InChI encoding, thereby minimizing experimental heterogeneity [DOI: 10.1093/nar/gky1075].

Importantly, ChEMBL’s data architecture is highly compatible with the objectives of this study. The database provides systematic coverage of the nuclear receptor superfamily, including ERα, with more than 17,000 annotated targets in its 2023 Release 33 version. It integrates data from binding assays, functional studies, and ADMET profiling, offering a robust foundation for our dual-phase modeling framework—activity prediction and ADMET evaluation [DOI: 10.1093/nar/gkad1004; DOI:10.1093/nar/gkw1074]. Moreover, ChEMBL’s periodic version updates and direct data deposition mechanisms (e.g., through the EUbOPEN chemical probe initiative) ensure that it continuously incorporates the latest experimental findings. This real-time updating prevents model development from being constrained by outdated datasets and enables our study to reflect the most current trends in ERα-targeted drug discovery.

Comment 2. A comparison with other feature selection methods would further validate the proposed DFFS approach.

Re: We sincerely thank the reviewer for the constructive suggestion regarding a comparative evaluation of the proposed DFFS method against other feature selection algorithms. A detailed explanation is provided below.

1. Experimental Design and Method Comparison

The DFFS method integrates three feature importance calculation mechanisms—MI, RF, and XGBoost—to obtain a final feature subset through weighted aggregation. This approach balances statistical correlation and the expressive power of nonlinear structural features. During feature selection, to ensure robustness and reproducibility, we implemented five-fold cross-validation and incorporated controlled randomness via RF’s bootstrap sampling and XGBoost’s multi-level feature subsampling mechanisms (colsample_bytree, colsample_bynode, and subsample) at both sample and feature dimensions. This design enhances the model’s resilience to data perturbations.

To evaluate DFFS’s effectiveness, we compared it against LightGBM models using feature subsets obtained individually from MI, XGBoost, and XGBoost-based SHAP values, with LightGBM serving as the downstream predictor, consistent with the original study.

Additionally, to examine deep learning–based feature generation for molecular representation, we introduced the self-supervised molecular language model ChemBERTa [DOI: 10.1186/s13321-024-00929-7] as a reference. Using Masked Language Modeling (MLM), ChemBERTa captures contextual semantic features from SMILES sequences. We generated 768-dimensional embeddings for each molecule via the pre-trained ChemBERTa-100M-MLM model on the HuggingFace platform. Using the same 4:1 train–test split, downstream regression was performed with LightGBM and multi-layer perceptron (MLP) models to compare the performance of DFFS-selected descriptors versus deep learning embeddings.

2. Results Analysis and Performance Comparison

Performance comparisons are summarized in Table 4. Models using individual MI, XGBoost, or SHAP feature selection achieved slightly lower test set performance (R² = 0.7388, 0.7269, 0.7449) compared to DFFS (R² = 0.7471), indicating that multi-strategy integration more effectively captures bioactivity-relevant features, improving both the representativeness of the feature subset and model generalization. While ChemBERTa embeddings provide strong nonlinear representational capacity, the downstream predictive performance on the current dataset (R² = 0.6338 for LightGBM, 0.6198 for MLP) was significantly lower than DFFS-LightGBM. This highlights that, 2D molecular descriptors combined with robust feature selection remain advantageous.

Table 4. Performance comparison of DFFS with other feature selection and molecular representation methods

| Feature Method | MRE | MSE | MAE | R² |
| --- | --- | --- | --- | --- |
| DFFS-LightGBM | **0.0775** | **0.4865** | **0.4959** | **0.7471** |
| MI-LightGBM | 0.0788 | 0.5026 | 0.5028 | 0.7388 |
| XGBoost-LightGBM | 0.0818 | 0.5254 | 0.5226 | 0.7269 |
| SHAP-LightGBM | 0.0811 | 0.4908 | 0.5183 | 0.7449 |
| ChemBERTa-LightGBM | 0.1032 | 0.7045 | 0.6507 | 0.6338 |
| ChemBERTa-MLP | 0.1011 | 0.7703 | 0.6365 | 0.6198 |

In summary, DFFS effectively captures key molecular bioactivity features while maintaining high reproducibility and stability, providing a reliable input foundation for subsequent QSAR modeling. These experimental procedures and comparative results have been systematically incorporated into the revised manuscript section “*Screening Model Based on Activity Prediction*” to strengthen the study’s scientific rigor and credibility.

Comment 3. The discussion could benefit from being more closely linked to previous studies, to better contextualize the results.

Re: We sincerely thank the reviewer for the constructive suggestion to more closely link our findings with existing research. We have framed the Phi coefficient correlation analysis and the SHAP-based feature interpretation results within the context of current ADMET and molecular pharmacology literature. All conclusions are data-driven, and the revised manuscript now includes additional mechanistic evidence.

1. Literature Association and Mechanistic Interpretation of ADMET Property Correlations

Our data analysis identified several significant correlation patterns: First, Caco-2 permeability negatively correlates with hERG channel blockade risk, suggesting that compounds with favorable intestinal absorption tend to have reduced structural propensity for cardiotoxicity [DOI: 10.1021/acs.jcim.1c00744; DOI: 10.1186/s12859-019-2814-5; DOI: 10.1039/C8MD00472B]. Second, Caco-2 permeability negatively correlates with CYP3A4 metabolism while positively correlating with HOB, reflecting an absorption–metabolism trade-off for highly permeable compounds [DOI: 10.1124/dmd.105.008672; DOI: 10.1016/j.pharmthera.2012.12.007; DOI: 10.7717/peerj.18636]. Third, hERG risk positively correlates with CYP3A4 substrate risk, indicating overlap in chemical space and emphasizing the need to consider multiple ADMET risks concurrently during drug development [DOI: 10.1016/j.ejmech.2020.112290; DOI: 10.1021/acs.jcim.1c00744]. Finally, micronucleus (MN) genotoxicity appears independent of other ADMET properties, suggesting that its mechanism primarily involves DNA damage and is not directly related to pharmacokinetics or enzyme/receptor interactions [DOI: 10.2174/092986706778201558].

2. Mechanistic Interpretation of Key Molecular Descriptors and Literature Comparison

SHAP analysis identified LipoaffinityIndex as a critical descriptor for predicting pIC50. When LipoaffinityIndex > 8, compounds typically exhibit stronger bioactivity. LipoaffinityIndex quantifies the affinity of compounds for lipid environments, closely related to membrane permeability and cellular uptake efficiency. The crucial role of lipophilicity in ERα antagonist activity has been well established. As an intracellular nuclear receptor, ERα antagonists must first traverse the cell membrane to reach their intracellular targets [DOI: 10.1038/39645]. Higher lipophilicity facilitates passive diffusion across the lipid bilayer, enabling effective intracellular exposure at lower extracellular concentrations, thereby enhancing apparent pIC50 values [DOI: 10.1016/s0169-409x(00)00129-0]. Recent studies on selective estrogen receptor modulators (SERMs) further support this trend: optimal lipophilicity balances membrane permeability with receptor-binding affinity [DOI: 10.1016/j.pharmthera.2017.12.012; DOI: 10.1158/1078-0432.CCR-15-0360]. Computational chemistry results also indicate that moderate increases in lipophilicity can significantly improve ERα antagonist cellular uptake and efficacy, with LogP values of 4–7 often achieving the best balance [DOI: 10.1021/acs.jmedchem.6b01355; DOI: 10.2133/dmpk.19.327].

MDEC-23 exhibits a clear threshold effect on inhibitory activity: when MDEC-23 > 20, its impact on pIC50 shifts from negative to positive. MDEC-23 characterizes the topological distance between secondary and tertiary carbon atoms, reflecting the spatial extension and flexibility of the molecular scaffold. Low MDEC-23 values generally correspond to compact, rigid frameworks, which enhance intramolecular stability but limit conformational diversity and induced-fit capability [DOI: 10.2174/1574884711308020006]. In contrast, higher MDEC-23 values indicate greater spatial extension and flexibility, allowing molecules to adopt multiple conformations during binding and better accommodate the dynamic receptor binding pocket. Structural biology studies show that the ERα ligand-binding pocket is relatively spacious and can accommodate ligands of varying geometries via an induced-fit mechanism [DOI: 10.1021/bi901149t; DOI: 10.1016/j.molcel.2015.01.025; DOI: 10.7554/eLife.12792]. Molecular dynamics simulations further reveal that molecules with higher MDEC-23 values generally possess greater scaffold extension, enabling interactions with key residues in both agonist and antagonist regions, thereby stabilizing the receptor in an antagonistic conformation by preventing helix H12 repositioning [DOI: 10.1016/j.drudis.2016.11.001; DOI: 10.3389/fphar.2018.00492]. Therefore, the threshold of MDEC-23 = 20 may reflect the minimal extension required for effective cross-domain interactions. This finding aligns with recent structure–activity relationship studies, which suggest that molecules with moderate flexibility and spatial extension are more likely to form stable ERα antagonist binding modes and effectively block coactivator recruitment [DOI: 10.1158/2159-8290.CD-17-1229; DOI: 10.1158/0008-5472.CAN-15-2357].

Comment 4. It is recommended to improve the readability and quality of figures.

Re: We sincerely thank the reviewer for the valuable suggestion regarding improving the readability and overall quality of the figures. We have redesigned and optimized several figures and supplemented them with more explanatory captions to enhance both visual clarity and academic rigor.

As shown in Fig 1, the revised figure, “Two-Stage Target Prediction Flowchart,” presents the two-stage predictive framework of our model in a more logical and hierarchical manner. Compared with the original version, the new figure clearly distinguishes the two core components: “Stage I – Activity Regression” and “Stage II – ADMET Classification,” with explicit annotations of data flow and modeling logic. In each stage, we added detailed descriptions of dataset partitioning and modeling procedures: the dataset was split into training and test sets at an 8:2 ratio, five-fold cross-validation was applied during training, and model hyperparameters were globally optimized using the Bayesian Tree-structured Parzen Estimator (TPE) algorithm to ensure reproducibility and transparency.

In Stage I – Activity Regression, the framework is further divided into “Optimal Model” and “Activity Screening.” The “Optimal Model” module illustrates the comparison among multiple tree-based ensemble models and identifies LightGBM as the final optimal model. The “Activity Screening” module explains the activity threshold logic: using the formula pIC50 = -log(IC50), compounds with pIC50 ≥ 6 (equivalent to IC50 ≤ 1 μM) are selected for subsequent ADMET prediction. In Stage II – ADMET Classification, the framework is similarly divided into “Stacking Model” and “ADMET Profiling.” The “Stacking Model” module shows a stacked ensemble architecture using simple models and tree-based ensemble models as base learners with Gaussian Naive Bayes (GNB) as the meta-learner. The “ADMET Profiling” module displays the five binary ADMET prediction outcomes (0/1) generated by the stacking model, with concise functional annotations to reinforce the logical consistency of the figure.

Fig 1. Two-Stage Target Prediction Flowchart

Furthermore, in the revised manuscript, we have extensively expanded and refined the sections on model performance evaluation and interpretability analysis. We supplemented both activity regression and ADMET classification metrics, provided the Phi coefficient calculation formula with explanations, and added mechanistic insights in the SHAP feature importance analysis to better interpret key feature contributions. We also added and optimized multiple figures to illustrate model performance and structural validation, including: LightGBM performance comparisons under different feature selection methods, sample distributions for each ADMET binary classification task, predicted activity and binding energy analyses for highly active compounds, and molecular docking and receptor–ligand interaction visualizations with ERα. These enhancements substantially improve overall readability and the precision of scientific communication.

Comment 5. It would be useful for the authors to include a dedicated section on the study’s limitations, discussing methodological constraints.

Re: We sincerely thank the reviewer for the valuable suggestion to include a section on study limitations. In the revised manuscript, we have added a discussion of the primary methodological limitations, including ADMET dataset imbalance, the two-dimensional nature of the feature space, and the lack of molecular dynamics validation.

First, regarding the issue of class imbalance in the ADMET binary classification datasets, although a certain degree of imbalance existed, the extent of the disparity was relatively limited. Considering that model training in this study places greater emphasis on data authenticity and representativeness, no data balancing techniques were applied. We also attempted to employ the Synthetic Minority Oversampling Technique (SMOTE) to mitigate potential bias arising from class imbalance; however, the improvement in model performance was marginal, indicating that this approach may have limited applicability under the current data scale and distribution characteristics. In future work, we plan to explore structure-based generative balancing strategies or incorporate cost-sensitive learning mechanisms to more systematically address the imbalance issue. Moreover, expanding the experimental dataset will further enhance the model’s representativeness and generalization capability within realistic drug discovery scenarios.

Second, our study currently relies solely on two-dimensional molecular descriptors for feature construction. We fully recognize the inherent limitations of this approach: while 2D descriptors offer computational efficiency and facilitate model interpretability, they do not directly capture molecular three-dimensional conformations, electronic distributions, or stereochemical information. These 3D factors are critical for reflecting stereoselective recognition and binding energy changes between molecules and biological targets. Future work will integrate 3D structure-based quantitative descriptors—such as molecular mechanics energies, molecular surface electrostatic potentials, and hydrophobicity distributions—together with deep learning-based feature extraction methods to establish a multidimensional representation framework with higher biological relevance and interpretability, thereby improving predictive accuracy and mechanistic rationality.

Finally, regarding the absence of molecular dynamics (MD) validation, although we have supplemented the revised study with molecular docking analyses to assess the plausibility of predicted results via static binding energy calculations, we explicitly acknowledge that MD simulations were not performed, representing a significant limitation. Compared to docking, which provides instantaneous conformational snapshots, MD simulations can track the dynamic behavior of ligand–receptor complexes over time, including conformational fluctuations, hydrogen bond stability, hydrophobic interaction rearrangements, and free energy variations, thereby more realistically reflecting binding mechanisms under physiological conditions. Future studies will incorporate MD simulations to more comprehensively evaluate the structural stability and binding energy evolution of key complexes, further enhancing the biological interpretability and dynamic reliability of our predictive models.

Comment 6. Although the metrics are defined, it would be helpful to explain what typical values represent for these metrics (e.g., what values indicate a good model in this context).

Re: We sincerely thank the reviewer for the valuable suggestion regarding typical reference ranges for model evaluation metrics. We provide a detailed explanation in revised paper. Referring to the published literature, the following typical indicators are emphasized and explained

For the activity regression prediction model, we employed four commonly used metrics: MRE, MSE, MAE, R². In this study, R² serves as the primary evaluation criterion, supplemented by the error-based metrics for comprehensive assessment. Previous studies indicate that in QSAR-based predictive models for ERα inhibitory activity, an R² ≥ 0.6 on the test set is generally considered indicative of reasonable predictive performance, sufficient to meet the scientific requirements for activity prediction and virtual screening [DOI: 10.1002/minf.201000061; DOI: 10.1039/C7RA10979B]. In our study, the optimal model achieved an R² of 0.7475, while the ChemBERTa transfer learning-based model reached R² = 0.6338, further confirming the robustness of our model.

For the ADMET classification prediction models, we evaluated performance using Accuracy, Precision, Recall, F1 score, and AUC. Values closer to 1 indicate better model performance. Regarding the lack of unified AUC thresholds for ADMET binary classification tasks in QSAR studies, breast cancer-related ADMET modeling studies provide a useful reference. For instance, a multi-algorithm ensemble QSAR model for predicting inhibitors of the breast cancer resistance protein BCRP/ABCG2 reported an optimal classifier achieving an AUC close to 0.95 on the test set, demonstrating high predictive accuracy and generalizability [DOI: 10.1186/s13321-020-00421-y]. Based on these benchmarks, the ADMET models in our study achieved AUC values exceeding 0.95, indicating predictive performance at an equal or superior level.

Comment 7. The abstract mentions that high pIC50 values indicate good binding affinity to the ERα receptor. However, it would be valuable to directly evaluate the binding affinity of the high-activity candidate compounds, for example through ΔG binding calculations or molecular docking studies. This would help validate and complement the bioactivity predictions by providing a quantitative measure of the ligand-receptor interaction.

Re: We sincerely thank the reviewer for the valuable suggestion regarding the validation of binding affinity for high-activity candidate compounds. In response, we have supplemented the revised manuscript with molecular docking experiments to quantitatively evaluate the plausibility of our model predictions from a structural perspective. The detailed description and newly added figures are as follows.

Fig 2. Predicted activity and binding energy analysis of high-activity compounds: Panels (a)–(d) correspond to the five top-ranked compounds.

Fig 3. Molecular docking and interaction analysis of high-activity compounds with ERα: Panels (a)–(d) correspond to Compound 1, showing its 3D binding conformation, 2D interaction diagram, electrostatic potential distribution, and docking binding energy map; panels (e)–(h) correspond to the other four high-activity compounds, illustrating the visualized ligand–protein interface interactions.

In the extended study, we selected an additional 50 compounds without annotated activity or ADMET profiles as an independent validation set, based on predictions from the DFFS-LightGBM model (SMILES input; 729 molecular descriptors calculated). Prediction results indicated that 14 compounds had pIC50 > 7, 30 compounds fell within pIC50 = 6–7, and 6 compounds had pIC50 < 6. The five compounds with the highest predicted activity are shown in Fig 2, with predicted pIC50 values of 8.0721, 7.5194, 7.4693, 7.4377, and 7.4263, respectively. Based on these results, the top five high-activity compounds were subjected to molecular docking with ERα. Docking results revealed that all five compounds exhibited binding free energies (ΔG) below −7.0 kcal/mol, indicating strong binding affinity. Notably, the compound with the lowest ΔG (−8.3 kcal/mol) corresponded to the highest predicted activity (pIC50 = 8.0721), showing a consistent trend and validating the model’s effectiveness and stability in identifying high-affinity ligands [DOI: 10.1016/j.phymed.2023.154813].

Furthermore, we conducted a systematic structural analysis and interaction feature visualization for Compound 1 (Fig.2), which exhibited both the highest predicted activity and the lowest binding energy, in complex with ERα (Fig 3, panels a–d). The analysis includes:

1. 3D binding conformation, illustrating the spatial complementarity of the ligand within the ERα active site and its interactions with key amino acid residues;
2. 2D interaction diagram (LigPlot⁺), quantitatively depicting hydrogen bonds, hydrophobic interactions, π–π stacking, and van der Waals contacts;
3. Electrostatic potential map, showing the complementary distribution of charged regions in the binding site and polar groups of the ligand;
4. Docking binding energies, presenting the affinities (in kcal/mol) of nine binding modes along with their corresponding root-mean-square deviation lower and upper bounds (rmsd l.b. and rmsd u.b.), where lower binding energies indicate more stable ligand–receptor interactions.

Additionally, the binding interface interactions of the other four high-activity compounds were visualized (Figure 3, panels e–h). Overall, all high-activity compounds exhibited stable, multi-point binding features with ERα. The binding energy results were highly consistent with the activity predictions, further validating the reliability of the DFFS-LightGBM model in molecular activity recognition and screening.

Comment 8. Information included on the different modeling techniques, QSAR and ADMET, is quite trivial and should be more detailed. To enhance the introduction of the computational section, it is recommended to provide a detailed discussion as reported in the literature. In order to facilitate a better understanding for readers, the following previous studies employing docking and molecular dynamic in this research area can be cited: [10.1002/jccs.202200195](https://doi.org/10.1002/jccs.202200195); [10.1016/j.molstruc.2022.133964](https://doi.org/10.1016/j.molstruc.2022.133964); [10.1016/j.jtusci.2015.04.007](https://doi.org/10.1016/j.jtusci.2015.04.007); [10.1021/acsomega.2c07585](https://doi.org/10.1021/acsomega.2c07585); [10.1002/slct.202301092](https://doi.org/10.1002/slct.202301092); [10.1016/j.chphi.2023.100361](https://doi.org/10.1016/j.chphi.2023.100361); [10.1155/2016/5137289](https://doi.org/10.1155/2016/5137289).

Re: We sincerely thank the reviewer for the valuable suggestion regarding the computational section. In response, we have supplemented and refined this part by including molecular docking analyses to strengthen the mechanistic interpretation of drug–target interactions. Following the reviewer’s advice, we referenced several recent high-quality studies to enrich the background and methodological discussion.

Specifically, three molecular docking studies were cited in the revised “*Introduction*” and “*Molecular Docking and Binding Mode Analysis*” sections [DOI: 10.1002/jccs.202200195; DOI: 10.1021/acsomega.2c07585; DOI: 10.1016/j.chphi.2023.100361]. These studies provided methodological references for our docking workflow, supporting the reliability of our computational predictions in assessing ligand–target interactions and binding stability.

1. Khedraoui et al. analyzed 32 dihydroflavanone derivatives using QSAR modeling and molecular docking to predict their affinity for Acetylcholinesterase (AChE), and molecular dynamics simulations confirmed the stability of the ligand–AChE complexes.
2. Nour et al. evaluated eugenol derivatives with DFT, docking, and molecular dynamics, showing moderate to high binding affinities against cholinesterase enzymes and stable complexes for the most promising dual inhibitors.
3. Daoui et al. screened cyclohexane-1,3-dione derivatives for c-Met inhibition using QSAR, docking, and ADMET modeling, with docking and MM-GBSA analyses identifying optimal scaffolds, and MD simulations validating the stability of lead compound–c-Met complexes.

In the revised manuscript, within the “*Screening Model Based on Activity Prediction*” section, we cited a recent study relevant to the application of principal component analysis (PCA) [DOI: 10.1016/j.molstruc.2022.133964]. Daoui et al. conducted an in silico study on pyridine derivatives as potential MALT1 allosteric inhibitors, using QSAR modeling, molecular docking, and MD simulations to optimize biological inhibitory activity. PCA and k-means clustering were applied to divide and optimize the training and test sets for QSAR model development, providing a methodological reference for our descriptor dimensionality reduction and dataset partitioning.
